# Comprehensive RNA analysis of CSF reveals a role for CEACAM6 in lung cancer leptomeningeal metastases

**DOI:** 10.1038/s41698-021-00228-6

**Published:** 2021-10-08

**Authors:** Yingmei Li, Dina Polyak, Layton Lamsam, Ian David Connolly, Eli Johnson, Lina Khav Khoeur, Stephanie Andersen, Monica Granucci, Geoff Stanley, Boxiang Liu, Seema Nagpal, Melanie Hayden Gephart

**Affiliations:** 1 Department of Neurosurgery, Stanford University School of Medicine, Stanford, CA USA; 2 Department of Biophysics, Stanford University School of Medicine, Stanford, CA USA; 3 Department of Biology, Stanford University School of Humanities & Sciences, Stanford, CA USA; 4 Department of Neurology & Neurological Sciences, Stanford University School of Medicine, Stanford, CA USA

**Keywords:** Cancer genetics, Cancer genetics, Molecular biology, Non-small-cell lung cancer

## Abstract

Non-small cell lung cancer (NSCLC) metastatic to the brain leptomeninges is rapidly fatal, cannot be biopsied, and cancer cells in the cerebrospinal fluid (CSF) are few; therefore, available tissue samples to develop effective treatments are severely limited. This study aimed to converge single-cell RNA-seq and cell-free RNA (cfRNA) analyses to both diagnose NSCLC leptomeningeal metastases (LM), and to use gene expression profiles to understand progression mechanisms of NSCLC in the brain leptomeninges. NSCLC patients with suspected LM underwent withdrawal of CSF via lumbar puncture. Four cytology-positive CSF samples underwent single-cell capture (*n* = 197 cells) by microfluidic chip. Using robust principal component analyses, NSCLC LM cell gene expression was compared to immune cells. Massively parallel qPCR (9216 simultaneous reactions) on human CSF cfRNA samples compared the relative gene expression of patients with NSCLC LM (*n* = 14) to non-tumor controls (*n* = 7). The NSCLC-associated gene, *CEACAM6*, underwent in vitro validation in NSCLC cell lines for involvement in pathologic behaviors characteristic of LM. NSCLC LM gene expression revealed by single-cell RNA-seq was also reflected in CSF cfRNA of cytology-positive patients. Tumor-associated cfRNA (e.g., *CEACAM6*, *MUC1*) was present in NSCLC LM patients’ CSF, but not in controls (*CEACAM6* detection sensitivity 88.24% and specificity 100%). Cell migration in NSCLC cell lines was directly proportional to *CEACAM6* expression, suggesting a role in disease progression. NSCLC-associated cfRNA is detectable in the CSF of patients with LM, and corresponds to the gene expression profile of NSCLC LM cells. *CEACAM6* contributes significantly to NSCLC migration, a hallmark of LM pathophysiology.

## Introduction

Patients with non-small cell lung cancer (NSCLC) are at high risk of developing leptomeningeal brain metastases (LM), where diffuse metastatic cancer growth on the surface of the brain and cranial nerves is rapidly fatal^1^. Despite an extremely poor prognosis (2–6 months)^2–4^ and increasing incidence, patients have few effective treatment options available^5^. Due to the diffuse nature of LM, surgical resection or biopsy are not feasible. The relative rarity of cases, poor prognosis, and lack of tissue available for research have stymied LM research to understand mechanisms of progression and develop new treatments.

We previously demonstrated that brain tumor-associated cell-free DNA (cfDNA) in cerebrospinal fluid (CSF) can be used to detect LM and track its response to therapy and relapse^6,7^. This method is reliable and clinically relevant, enabling the detection of tumor-specific mutations in CSF that direct therapy (e.g., *EGFR, BRAF*) even in patients with no measurable systemic disease. Yet, DNA mutations are only one subset of cancer aberrations; investigating the gene expression profiles of LM would allow for a more complete understanding of the disruptive systems LM utilizes for disease progression. We sought to close this gap through the study of tumor-associated cellular and cell-free mRNA levels in the CSF of patients with NSCLC LM.

**Fig. 1:**
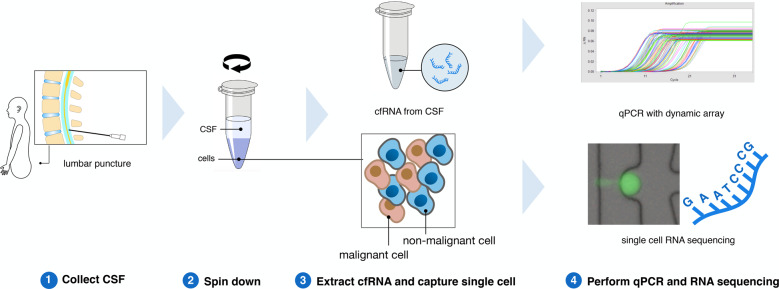
RNA analysis pipeline for processing human cerebrospinal fluid. Fresh CSF samples were collected from patients and then underwent centrifugation to separate cells and supernatant. cfRNA was extracted from the supernatant and analyzed by qPCR dynamic array. Single cells from CSF were captured by a microfluidic chip and prepared for single-cell RNA sequencing.

We overcame the critical lack of tissue samples available for research by adapting and combining two innovative techniques, and applying these directly to human LM CSF and non-tumor control samples. A comprehensive RNA analysis of NSCLC patient CSF samples was accomplished via single LM cell capture, RNA sequencing, transcriptome analysis (scRNA-seq), and cell-free RNA (cfRNA) qPCR multiplexed microfluidic analyses (Fig. 1). These data showed cfRNA levels reflected the elevated expression of single NSCLC LM cells, identifying genes both known (e.g., *MUC1*) and novel (e.g., *CEACAM6*) for NSCLC progression. Elevated levels of *CEACAM6* in cfRNA of patients with NSCLC LM were not present in non-LM controls. We identified a new role for *CEACAM6* in NSCLC, finding elevated expression correlated with cellular migration, a key component of LM pathology. We anticipate that further study of the scRNA and cfRNA profiles of LM will unveil mechanisms of disease progression, with projected translational benefits.

## Results

### Parallel qPCR of cfRNA in patient CSF identifies NSCLC LM-specific gene expression

**Fig. 2:**
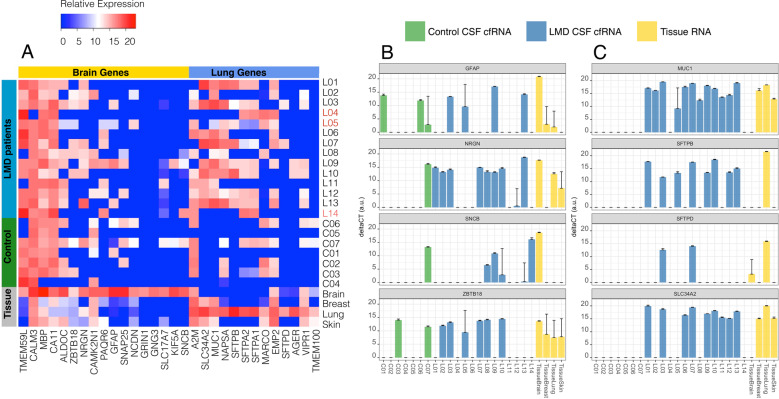
Relative expression of tissue-specific genes in cfRNA from CSF. **A** A panel of lung- and brain-specific gene expression across all samples. All LM samples were cytology-positive except L04, L05, and L14 (shown in red). **B** A subset of brain-specific genes in some patients showed elevated expression, however, a couple of control patients also had detectable levels of expression. **C** Lung-specific genes, however, were only detected in patients with LM, compared to no expression of these genes in the CSF of patients without metastatic disease.

To identify NSCLC LM-associated cfRNA in patient CSF, we created a panel of lung and brain-specific genes for parallel qPCR (Supplementary Fig. 1). These tissue-specific genes were chosen based on information in the Genotype-Tissue Expression (GTEx) database (obtained from the GTEx Portal on 6/30/2017; https://www.gtexportal.org/home/). Briefly, the top 100 expressed genes in each major organ (e.g., brain, lung, skin) were selected, and genes that overlapped between these organs were excluded. Fourteen CSF cfRNA samples (L01–L14) from patients with NSCLC LM were compared with seven control patients (C01-C07; Fig. 2A); control patients included patients without cancer, or cytology-negative patients with NSCLC. The numbers of detected genes from the 96-gene panel did not significantly differ between control patients, cytology-negative patients, and cytology-positive patients (Supplementary Fig. 2).

The expression level of brain- and lung-specific genes for each sample are shown in Fig. 2A. RNA expression of these genes in bulk tissue acted as a positive control for lung and brain specificity (Fig. 2A, bottom four rows). Several brain-specific genes (*GFAP, NRGN, SNCB, ZBTB18*; Fig. 2B) were more frequently detected in LM samples. Lung-specific genes, such as *MUC1, SFTPB, SFTPD, SLC34A2*, were only detected in LM CSF samples (Fig. 2C).

### Cell-free RNA in CSF reflects the gene expression profiles of single NSCLC LM cells

**Fig. 3:**
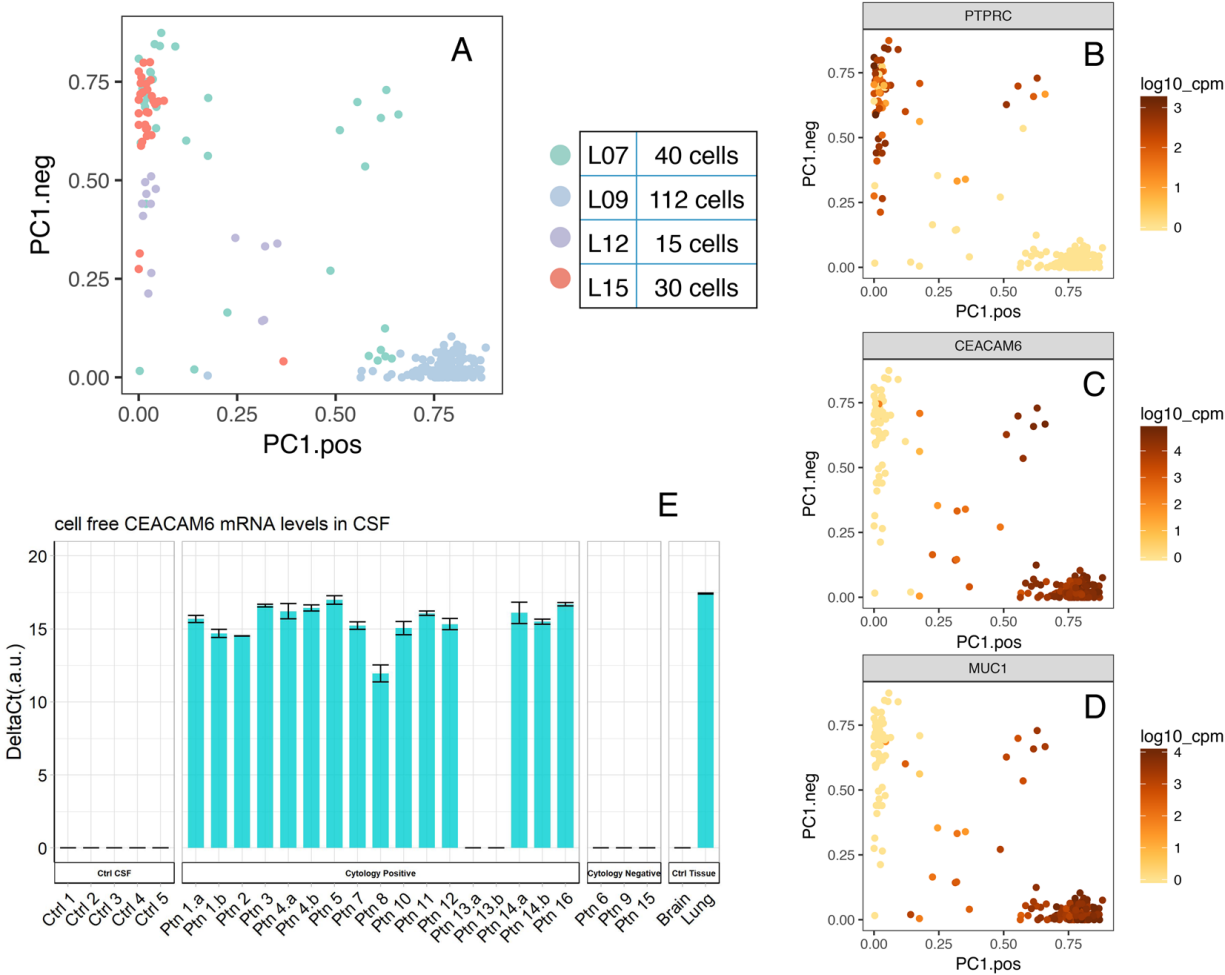
Single-cell RNA-seq of human cells in cerebrospinal fluid. **A** Robust rPCA representation of all single cells included in this study (*n* = 197) from 4 patients. Cells are colored by sample ID. **B**–**D** Expression of characteristic cell-type-specific genes overlaid on the robust rPCA plot (*P* < 2 × 10^−16^, *t*-test). **E** Cell-free *CEACAM6* RNA level in CSF validated by qPCR. Results are shown as the mean ± standard deviation (SD) performed with three technical replicates.

CSF from patients with LM contains a variable and an unpredictable number of cells, from zero to millions^8^. Moreover, the clinical procedures to access CSF (e.g., lumbar puncture, ventricular access) inevitably introduce peripheral blood contamination. To investigate individual LM cells in CSF, we removed the red blood cells with lysis buffer, and all the other cells were loaded into a microfluidic chip for capture (Supplementary Fig. 3). One hundred and ninety-seven cells were sequenced from four cytology-positive patients (for patient information see Supplementary Table 1, for cytology reports see Supplementary Table 2). Each individual cell had 1–5 (median = 3.2) million paired-end reads, mapping rate ranging from 12.6 to 70% (median = 44.3%). The detailed bioinformatic pipeline can be found in the methods section. Gene count data underwent iterative robust Principal Component Analysis (rPCA). To identify NSCLC LM cells separate from contaminating blood cells we inferred the cell identities by determining significantly differentially expressed genes in each cluster (Fig. 3A). Genes whose expressions were specific to each cluster are shown in Fig. 3B–D. High expression of *PTPRC* identified white blood cells (WBCs) (Fig. 3B), while high expression of *CEACAM6* (Fig. 3C) and *MUC1* (Fig. 3D) were found in the NSCLC LM cells (*P* < 2 × 10^−16^, *t*-test). More characteristic genes can be found in Supplementary Fig. 4.

*MUC1* was present in our lung tissue-specific cfRNA panel and detected in NSCLC LM CSF (Fig. 2C); *CEACAM6* was not included in our initial tissue-specific cfRNA panel. To find out whether *CEACAM6* could be detected in CSF cfRNA of patients with NSCLC LM, we performed qPCR (primers from ThermoFisher Scientific) on a validation cohort of patients suspected to have NSCLC LM and healthy control patients (Fig. 3E and Supplementary Table 3). *CEACAM6* transcripts were detected in 15 out of 17 cytology-positive patients with NSCLC LM (Supplementary Table 3), while no *CEACAM6* cell-free mRNA was detected in CSF of cytology-negative (*n* = 3) or healthy control (*n* = 5) CSF samples. Using positive cytology as a true positive, the sensitivity of *CEACAM6* detection was 88.24% with a specificity of 100%, the positive prediction value of 100%, and a negative predictive value of 60% (Supplementary Table 4). Of note, one of the false-negative CSF samples (Ptn 13a. and b.) showed extensive hemolysis due to a prior intraventricular hemorrhage (Supplementary Fig. 5). Contamination of CSF with blood products has been previously reported to be a major inhibitory factor of polymerase chain reaction and may explain the false-negative lack of *CEACAM6* transcript detection in sample number Ptn 13^9,10^.

### *CEACAM6* promotes the migration of lung cancer cells in vitro

**Fig. 4:**
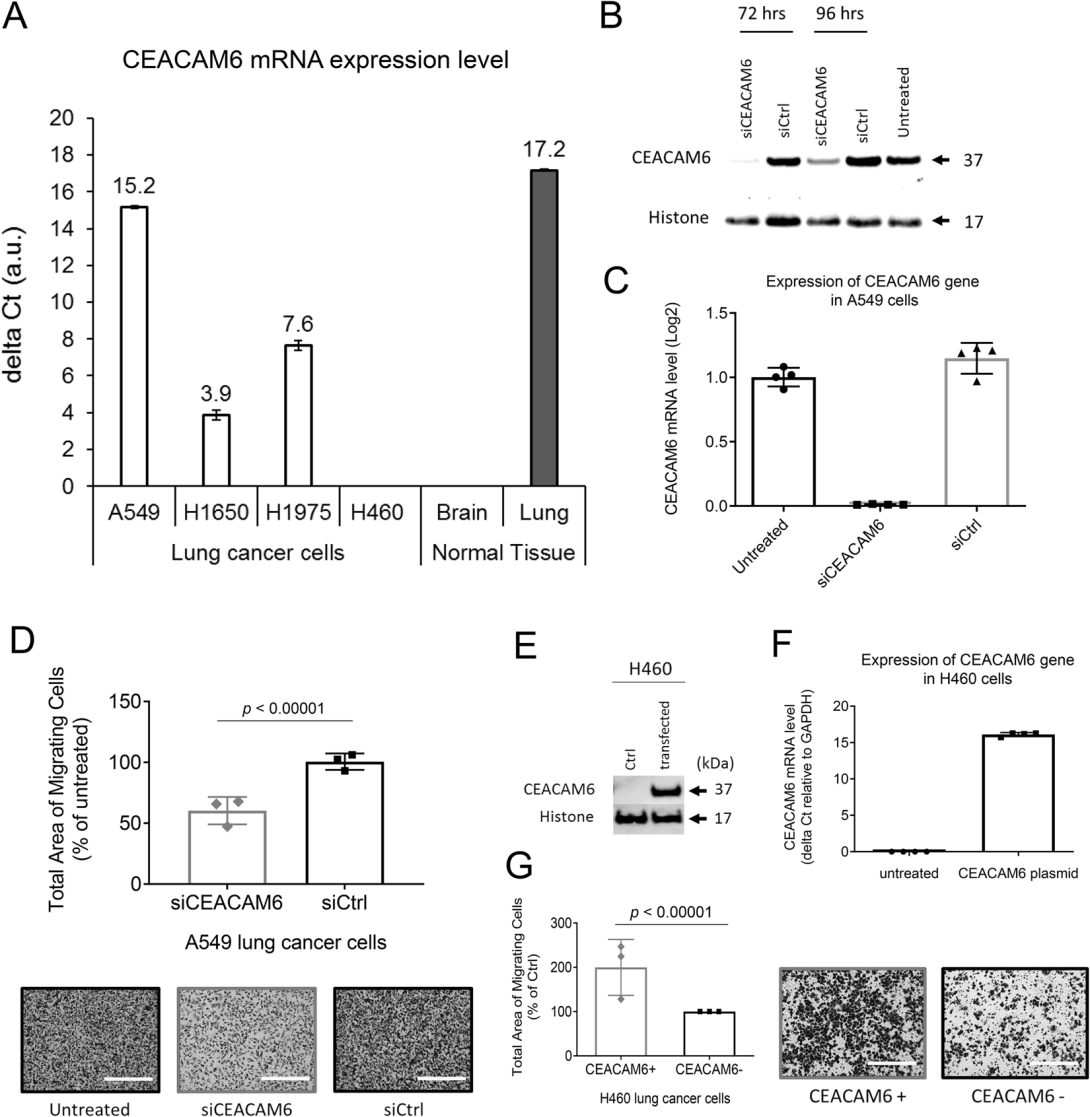
CEACAM6 mediates the migration of NSCLC cells. **A** A549 lung adenocarcinoma cells natively expressed high levels of *CEACAM6* as opposed to H460 cells that showed undetectable levels of *CEACAM6*, as measured by qPCR. A549 cells were treated for 72 h with siCEACAM6 failed to migrate toward FBS. **B** Western blot analysis confirmed CEACAM6 knockdown in A549 cells following treatment with siRNA. **C** qPCR analysis confirmed *CEACAM6* knockdown in A549 cells following 72 h knockdown with siRNA. **D** Quantitative analysis and representative images of decreased migration of A549 cells following knockdown of *CEACAM6*, as compared with siCtrl-treated cells (normalized to untreated cells). **E** Western blot and **F** qPCR confirmed elevated *CEACAM6* levels in H460 cells following plasmid transfection. **G** Quantitative analysis and representative images showed increased cell migration following overexpression of CEACAM6, as compared with H460 cells. Student’s *t*-test was used to evaluate the statistical significance of the difference between groups in **D** and **G**. Scale bar = 400 µm in **D** and **G** images. Results are shown as the mean ± standard deviation (SD) of three independent assays.

Diffuse migration and invasion are key hallmarks of NSCLC LM, a phenotype that also makes it particularly resistant to treatment. Expression of *CEACAM6* in pancreatic, gastric, and other cancers was previously shown to predict poor survival and advanced metastatic progression^11–14^. To study the role of *CEACAM6* in NSCLC we screened lung cancer cell lines for their expression of *CEACAM6* (Fig. 4A). A549 lung adenocarcinoma cells natively express high levels of *CEACAM6*, and no native expression of *CEACAM6* was detected in H460 cells or brain tissue. We treated A549 lung adenocarcinoma cells (natively high levels of *CEACAM6* expression) for 72 h with 100 nM siCEACAM6 or siCtrl. Knockdown of *CEACAM6* was confirmed via western blot and qPCR (Fig. 4B–C). FBS-facilitated migration of A549 cells was inhibited by 40% with *CEACAM6* siRNA as compared to control siRNA-treated cells (Fig. 4D). Having confirmed that the knockdown of *CEACAM6* in native high expressing cells resulted in decreased NSCLC migration, we then hypothesized that overexpression in cells that did not natively express *CEACAM6* would lead to increased migration. Therefore, H460 lung cancer cells were transfected with *CEACAM6* plasmid; we observed a 2-fold increase in migration, as compared to control cells (Fig. 4E–G).

## Discussion

Given that LM cannot be biopsied and the number of cancer cells in CSF is so few and variable, we needed a method to investigate the genes active in NSCLC LM. We previously showed primary tumor driver mutations (e.g., *EGFR, BRAF*) are also present in LM, and reliably detected in CSF cfDNA of patients with brain metastases^6,7,15^. Expanding to cfRNA, as a proof of principle we hypothesized that NSCLC LM cells would still preserve some RNA expression features of the primary lung cells from which they originated. We sought to detect tumor-associated cfRNA in patient CSF when NSCLC LM was present, as the normal CSF should have no such lung-specific gene transcripts. Results from a tissue-specific, massively parallel qPCR (9216 simultaneous reactions) of cfRNA from patient CSF revealed that lung-specific gene expression from NSCLC patients was more common than in control CSF samples (Fig. 2A). While brain-specific RNA was sporadically detected in the control and LM patients (Fig. 2B), these data suggest future studies may query the brain or immune-specific response to the presence of LM by testing the cfRNA component of CSF. Among the lung-associated genes, *SFTPB* and *SFTPD* were highly specific to the lung, as demonstrated by the four tissue RNA samples (Fig. 2C), and they were solely found in lung cancer LM patients’ CSF. This result suggested a potential clinical application of cfRNA as a diagnostic tool for patients with suspicion of NSCLC LM. Only a subset of tissue-specific or cancer-related genes included in this panel (Supplementary tabular data_Gene panel; Supplementary Fig. 1) showed statistically significant differences between patients with cytology-confirmed NSCLC LM and non-cancer controls. Our finding of *MUC1* expression in patient CSF is consistent with prior reports of using *MUC1* to identify NSCLC circulating tumor cells^16^. The method of cfRNA detection could be a valuable test of response to therapy and relapse by evaluating cfRNA cancer gene expression and cytology in parallel, without the burden of the extra sampling. To successfully translate the use of cfRNA as a diagnostic or scientific tool, a large, multi-institutional cohort of samples should be tested, given the relative rarity of LM. To enable this collaboration, an effort to standardize the approach to sample collection and processing for cfRNA analysis from CSF is currently ongoing. Detection of malignant cells on cytologic examination of the CSF is the diagnostic gold standard for LM, however, malignant cells in the CSF are scant, and even when present, information about tumor biology cannot be derived. Even 20cc of CSF (15% of the total CSF volume) may render no malignant cells, or only one or two atypical cells, which leads to multiple repeat lumbar punctures in order to confirm the diagnosis. This makes the capture of LM cells from CSF very difficult and, when achieved, results in a low overall number of cells. A distinct advantage of sampling the CSF, however, is that the CSF is intrinsically acellular and so reduces the background signal for captured CSF circulating tumor cells or cell-free nucleic acids; this is in contrast to blood circulating tumor cells which are mixed with a large number of contaminating normal blood cells and cell-free nucleic acids from the entire body. A subset of the tissue-specific cells in our gene panel found in NSCLC LM CSF but not in normal controls, have been previously described to also play a role in cancer. For example, *MUC1* is often overexpressed in cancer and plays a key role in cancer progression, increasing the bulkiness of the glycocalyx to help cells survive anoikis^17,18^. We sought to extend our testing of NSCLC LM genes within CSF beyond tissue specificity, and needed to validate that NSCLC-associated cfRNA was reflective of the cancer cells in CSF. Given the frequent contamination by peripheral blood of the lumbar or ventricular access technique to release CSF, we required single-cell resolution to identify and investigate NCSLC LM in CSF. In addition to validating elevated levels of *MUC1* expression in NSCLC LM cells (Fig. 3D), single-cell RNA-seq of NSCLC LM showed high expression of *CEACAM6. CEACAM6* is a multi-functional glycoprotein that is often overexpressed in epithelial malignancy, correlating with adverse clinical outcomes^19^. Given the previously described link in NSCLC between *MUC1* expression and poor overall survival^20,21^, we chose to focus on *CEACAM6* in NSCLC LM. We first confirmed the detection of *CEACAM6* in the cfRNA component of a validation cohort of 21 patients (Supplementary Tables 1 and 3). Of these, five were control CSF in patients without cancer, and three were patients with NSCLC who at the time of sample collection were suspected to have LM. With the exception of one patient with significant hemolysis (Supplementary Fig. 5), all of the patients with cytology-positive NSCLC LM had detectable *CEACAM6* cfRNA expression, not present in the control samples. Of note, we previously showed a higher frequency and earlier onset of LM in patients with EGFR mutations^15^, and the predominance of patients with EGFR mutations was also present in our patient cohort (Supplementary Table 1). The detection of *CEACAM6* in tumor-associated cfRNA, but not in controls, was true despite variable clinical treatments (Supplementary Table 3). These data suggested a role for *CEACAM6* as a diagnostic marker and led us to investigate its role in the aggressive phenotype of NSCLC LM. The expression and function of *CEACAM6* in LM have not been previously described, likely due to the lack of accessible tissue or sensitive techniques to investigate human CSF mRNA levels. Our scRNA sequencing demonstrated that LM tumor cells had elevated levels of *CEACAM6* expression, present across cfRNA samples tested via qPCR in a validation cohort. Cancer cells propagate to distant organs to form metastases through a series of complex and stochastic events. These cells are either inherently metastatic or acquire traits through treatment pressure that allow them to invade distant organs. One key feature of cancer metastases is the cells’ ability to migrate. CEACAM6 protein functions by organizing tissue architecture and regulation of signal transduction to promote cell adhesion and invasion^22^. We tested two NSCLC cell lines with different baseline levels of *CEACAM6* expression to demonstrate the effect on migration capacity. H460 cells have low metastatic capacity^23,24^, however, overexpression of *CEACAM6* conferred these cells the ability to migrate. Conversely, when *CEACAM6* expression was reduced in the highly metastatic A549 cells^25^, migration was likewise significantly inhibited (Fig. 4D). Future multi-institutional study of matched human tumor tissue (primary lung, solid parenchymal brain metastases, and LM), and genetically modifiable in vivo mouse models of LM, will allow us to further determine how and under what treatment pressure *CEACAM6* promotes brain-trophic metastases. In conclusion, we have developed new CSF-based approaches to study LM in NSCLC patients using sensitive, high throughput techniques. We have identified lung and NSCLC-specific genes in cfRNA from CSF of patients with LM, which could be used as a diagnostic and scientific tool. We used this technique to suggest that *CEACAM6* plays a key role in NSCLC migration. Future studies of LM and brain-associated gene expression profiles in the cfRNA component of CSF will help to further elucidate the complex mechanisms of disease progression, and suggest novel treatment strategies.

## Methods

### Clinical sample collection and processing

Human CSF samples were collected from patients with or without cancer (controls) through a Stanford University Institutional Review Board-approved protocol. As a proof of concept case series, sample availability depended upon patient consent, volume obtained, and quality of the sample; patients were not specifically recruited for this study. LM CSF samples were obtained either from a standard-of-care lumbar puncture (LP) or ventriculostomy. Patients either required CSF access for diagnosis (e.g., equivocal MRI and/or concerning symptoms of LM), or therapeutic treatment of increased intracranial pressure. Only CSF samples in excess of what was required for clinical-pathological diagnosis were utilized in this study; as a pilot trial, no additional, invasive procedures were performed. Only CSF not required for clinical analyses were released to the laboratory. Treatments prior to the diagnosis of LM, as defined by positive cytology, included chemotherapy, targeted therapy, immunotherapy, and/or radiotherapy (Supplementary Tables 1, 3 and 5).

A minimal volume of 2 mL in a non-hemolyzed sample was required to process the sample. Given very low levels of nucleic acids in CSF and high rates of degradation of RNA, in our experience lower volumes have not been successful in extracting cfRNA. It is important to note that the scientists were “blinded” to the cytology results at the time of sample collection and processing. Given the high expense of single-cell RNA-seq, samples were first inspected via microscopy for the presence of cancer cells prior to further processing.

CSF was centrifuged (1000 × *g*, 10 min) to separate the cell pellet and supernatant within 1 h of collection. The cell pellet and supernatant components were transferred to separate tubes and stored at −80 °C until ready for RNA extraction. Cell-free RNA from 2 mL CSF was extracted using the Plasma/Serum Circulating and Exosomal RNA Purification Kit (Slurry Format) (Cat. 42800; Norgen Biotek Corp., Ontario, Canada), followed by a DNA removal step with Baseline-ZERO™ DNase (Epicentre, Lucigen Corporation, Middleton, WI), and cleaned up using RNA Clean and Concentrator Kits^TM^ (Zymo Research, Irvine, CA). RNA was then aliquoted and stored at −80 °C.

### Informed consent procedure

A researcher reviewed the consent with each patient or their legally authorized representative in a private room. The patient voluntarily signed the consent prior to any study procedures and after time was allotted so that all of the patient’s questions could be asked and answered. The patient was informed that they could withdraw from the trial at any time and their care could fully continue without prejudice. A copy of the signed consent was given to the patient and documentation of the patient’s consent was filed in their medical chart. If the patient’s primary language was not English, an interpreter and short-form documentation were provided per standard protocol.

### Parallel qPCR on targeted genes and analysis of Ct value

Cell-free RNA from 2 mL CSF was extracted and any contaminating DNA removed using the extraction processes as described above. Tissue-specific genes were chosen based on information from the GTEx database. Briefly, the top 100 expressed genes in each major organ (e.g., brain, lung, skin, blood) were selected, and genes that overlapped between these organs were excluded. PCR primers were designed by Fluidigm Delta Gene team (Fluidigm Corporation, San Francisco, CA). Extracted cfRNA was pre-amplified using the CellsDirect™ One-Step qRT-PCR Kit (Invitrogen™, ThermoFisher Scientific, Waltham, MA). Nineteen cycles of PCR were conducted, and the excessive primers were removed by Exonuclease I (New England Biolabs^®^, Ipswich, MA). To increase the dynamic range and accuracy of later qPCR steps, serial dilutions (5-, 10-, 20-fold) were performed on the cleaned PCR products, and each dilution was prepared in duplicate. Sample and targeted gene assays were loaded onto 96 x 96 dynamic array chips (Fluidigm) according to the manufacturer’s protocol. Bulk tissue RNA (Agilent Technologies, Santa Clara, CA) served as positive controls. The delta-Ct values of each gene with respect to the reference gene *ACTB* were calculated across all cfRNA samples and were compared with control patients. The delta-Ct method was described in a previous publication^26^. Briefly, delta_Ct(gene A) = raw_Ct (ACTB) − raw_Ct (gene A). The relative expression was adjusted by adding 20 universally to all delta-Ct values to have a positive value.

### Single-cell sequencing and data analysis

Cytology-positive CSF samples (*n* = 4) underwent single-cell capture by the Fluidigm^®^ C1™ system (Fluidigm). Red blood cells (RBCs) were lysed by the ACK Lysing Buffer (Life Technologies, ThermoFisher Scientific), followed by PBS washing. Cell suspension was loaded to the C1™ Single-Cell mRNA Seq IFC following the manufacturer’s protocol. The chip was taken to microscopic imaging after cell capture, and only cDNA from chambers with single live cells (Supplementary Fig. 3) were selected for sequencing library preparation. Pre-amplified cDNA generated from C1 was harvested and analyzed using the Fragment Analyzer™ Automated CE System (Advanced Analytical Technologies, Inc., Agilent). Only cells that showed nominal signs of RNA degradation and had a concentration higher than 0.05 ng/µL were selected for library preparation. The sequencing library was prepared using Nextera XT DNA Library Preparation Kit (Illumina) as described in the Fluidigm C1 protocol, and sequenced with 2 × 150 paired-end reads on a NextSeq mid-output kit (Illumina Inc., San Diego, CA). FastQC (version 0.11.4; http://www.bioinformatics.babraham.ac.uk/projects/fastqc/) was used for sequencing quality assessment^27^. Reads were then aligned to the human (hg19) transcriptome using Bowtie software (version 2.2.7)^28^ with splice junctions being defined in a Gene Transfer Format file (obtained from the University of California, Santa Cruz). Expression at gene level was determined by calculating reads per kilobase per million aligned reads (FPKM) as well as raw count using RSEM software version 1.2.30 (http://deweylab.github.io/RSEM/)^29^. After cells expressing fewer than 1000 genes were excluded (Supplementary Fig. 6), count data underwent iterative robust principal component analysis (rPCA) using the ROBPCA algorithm, which was previously shown to improve the separation of subtypes in scRNA-Seq analysis^30–32^. Fifteen principal components were specified. Analogous to gating in flow cytometry, sub-groups revealed by the initial round of rPCA were isolated and underwent another round of rPCA to further characterize heterogeneity in gene expression. Genes having the highest Pearson correlation coefficient with selected principal components were plotted and then colored by the common logarithm of the counts per million (CPM). Principal components used to stratify cells in this manner were checked for correlation with the number of expressed genes. Iterative rPCA analysis was conducted using R: A Language and Environment for Statistical Computing version 3.4.1 (R Foundation for Statistical Computing, Vienna, Austria; https://www.R-project.org).

### Reagents and cell culture

H460, H1650, and H1975 lung cancer cells were cultured in RPMI supplemented with 10% FBS. A549 lung cancer cells were cultured in DMEM supplemented with 10% FBS. All cells were maintained at 37 °C with 5% CO_2_. All cell lines were purchased from the American Type Culture Collection (Manassas, VA).

### Manipulation of *CEACAM6* gene expression in A549 and H460 cells

In vitro knockdown of the *CEACAM6* gene was achieved by the treatment of A549 cells with 250 nM of *CEACAM6*-specific small interfering RNA (siRNA) oligos, and non-targeting control siRNA (ON-TARGETplus SMARTpool and ON-TARGETplus Non-targeting Pool, accordingly, Dharmacon, Lafayette, CO, USA) for 72 h. To achieve overexpression of CEACAM6 protein in H460 cells, cells were transfected with full-length ready-to-use *CEACAM6* cDNA (Sino Biological Inc, Beijing, China) using Lipofectamine™2000 (Invitrogen™) according to the manufacturer’s protocol.

ON-TARGETplus SMARTpool siRNA CEACAM6 sequences:

GAUCACAGUCUCUGGAAGU, GAACAUGGCUAAAUACAAU GAGGGUAACUUAACAGAGU, CUACAUACUCCAACUGAAA

ON-TARGETplus Non-targeting Pool sequences:

UGGUUUACAUGUCGACUAA, UGGUUUACAUGUUGUGUGA, UGGUUUACAUGUUUUCUGA, UGGUUUACAUGUUUUCCUA

To quantify the *CEACAM6* mRNA and protein level in lung cancer cells, A549 cells were collected 72–96 h following incubation with siCEACAM6, siCtrl or DMEM media. H460 and H460/CEACAM6 cells were incubated in Hygromycin-free DMEM media for 4 days prior to collection. Total RNA was extracted from pelleted cells using RNeasy mini Kit (QIAGEN Inc., Germantown, MD) as per manufacturer’s protocol, followed by a DNA removal step with Baseline-ZERO™ DNase (Epicentre, Lucigen Corporation, Middleton, WI), and cleaned up by RNA Clean and Concentrator KitsTM (Zymo Research, Irvine, CA). RNA samples were eluted in 30–50 µL of nuclease-free water and stored at −80 °C for future use. The PCR master mix was based on TaqMan^®^ RNA-to-Ct™ 1-Step Kit (ThermoFisher Scientific, Waltham, MA), each sample was analyzed in quadruplicates. Primers and probes were used at concentrations of 150 and 250 nM per reaction, respectively. *CEACAM6* target gene was labeled with FAM probe and run together with *GAPDH* as a reference gene (probe labeled with HEX) in the same qPCR reaction on each individual sample.

For H460 cells, relative expression is calculated using the delta-Ct method using the following equations: ∆Ct (*CEACAM6*) = Ct (*CEACAM6*) − Ct (*GAPDH*); relative expression was adjusted by subtracting 20 universally to all delta-Ct values (relative expression = 20 − ∆Ct). For A549 cells, relative expression was calculated by the double delta-Ct method. Delta-Ct value of each sample condition was calculated as described above. Then, ∆Ct (untreated) was subtracted from ∆Ct (siCEACAM6) or ∆Ct (siCtrl) and the values were converted to express fold of change using 2 − ∆∆Ct equation. Primer sets to target *CEACAM6* (Hs00366002_m1) gene were obtained from ThermoFisher Scientific and to target *GAPDH* gene (Integrated DNA Technologies Inc., Coralville, IA). The qPCR reactions were carried out using the CFX96 Touch Real-Time PCR Detection System (Bio-Rad Laboratories, Hercules, CA).

*GAPDH* forward primer 5′-GAACGGGAAGCTTGTCATCAA-3′

*GAPDH* reverse primer 5′-ATCGCCCCACTTGATTTTGG-3′

### CEACAM6 protein detection

Cells were lysates in 1% SDS lysis buffer (10 mM Tris, pH7.4, 1% SDS, 10% glycerol, 75 mM NaCl) with cOmplete™ EDTA-free protease inhibitor cocktail (Sigma-Aldrich, St. Louis, MO). For enzymatic deglycosylation of CEACAM6, 10–20 μg of samples were digested with 500 units of recombinant endoglycosidase PNGase F (New England Biolabs^®^ Ipswich, MA) in 1% Nonidet P-40 and GlycoBuffer 2. Samples were incubated for 1 h at 37 °C after denaturation at 100 °C for 10 min and mixed with an equal volume of sample buffer. Protein (10 µg) was separated on 4–20% Tris-HCl gel then transferred onto nitrocellulose membrane using TransBlot^®^ Turbo™ RTA Transfer Kit (Bio-Rad Laboratories). Western blots were probed with antibodies against CEACAM6 9A6 (1:400, #MA1-17765; ThermoFisher Scientific) and Histone H2B (1:10,000, #07-371; Sigma-Aldrich). All blots derive from the same experiment and were processed in parallel.

### FBS-induced A549 and H460 cell migration assay

Cells migration assay was performed using modified 8 μm Boyden chambers by using CorningTranswell^®^ filters (Sigma-Aldrich, St. Louis, MO). A549 cells 72 h following incubation with siCEACAM6, siCtrl, or untreated were collected by trypsin, washed with serum-free media, and resuspended to a concentration of 50,000 cell/100 µL. H460 and H460/CEACAM6 cells were collected with trypsin, washed in serum-free media, and resuspended to a concentration of 200,000 cell/100 µL. Cells then were added to the upper chamber of the transwell (A549 50,000 cells/well, H460 200,000 cells/well) in serum-free DMEM or RPMI, respectively. Two hours later, cells were allowed to migrate to the 10% FBS-containing lower compartment of the chamber for 24–30 h. Cells on the upper part of the membrane were removed with a cotton swab, while migrated cells on the counter-side of the membrane were fixed and stained by immersion into crystal violet solution (1% w/v in 95% methanol). Stained cells were imaged using EVOS FL inverted microscope by ×10 objective. Four representative fields per well were imaged, three wells were examined for each condition, and the experiments were conducted in triplicates.

Quantification of migrated cells from captured images was counted using NIH ImageJ software (https://imagej.nih.gov/ij/). Percent of migrated A549 cells were normalized to untreated cells migrating toward FBS-containing media (no siRNA transfection). Percent of migrated H460/CEACAM6^+^ cells was normalized to migrated H460 cells. Results are shown as the mean ± standard deviation (SD) of three independent assays.

### Reporting summary

Further information on research design is available in the Nature Research Reporting Summary linked to this article.

## Supplementary information

Supplementary Information

Reporting Summary

## Data Availability

Gene count data can be found at the link https://figshare.com/account/home#/projects/78399. In addition, data will be added to the publicly available website: www.LMDseq.org. Sequencing data were uploaded to the Sequence Read Archive (SRA) database of the National Center for Biotechnology Information (NCBI) (https://www.ncbi.nlm.nih.gov/sra/), with the BioProject ID accession number: PRJNA754687. The non-sequencing data and materials are available from the corresponding author on reasonable request.

## Code availability

Analysis script was uploaded to Github and can be found using the link below: https://github.com/gmstanle/leptomeningeal-metastases-scRNseq.
