# Supplementary material for: Comprehensive RNA analysis of CSF reveals a role for CEACAM6 in lung cancer leptomeningeal metastases
Source: NPJ Precis Oncol. 2021 Oct 8;5:90. doi: 10.1038/s41698-021-00228-6 (PMC8501028; doi:10.1038/s41698-021-00228-6)
Supplement: Supplementary file 1 — Supplementary Information [file 41698_2021_228_MOESM1_ESM.pdf]

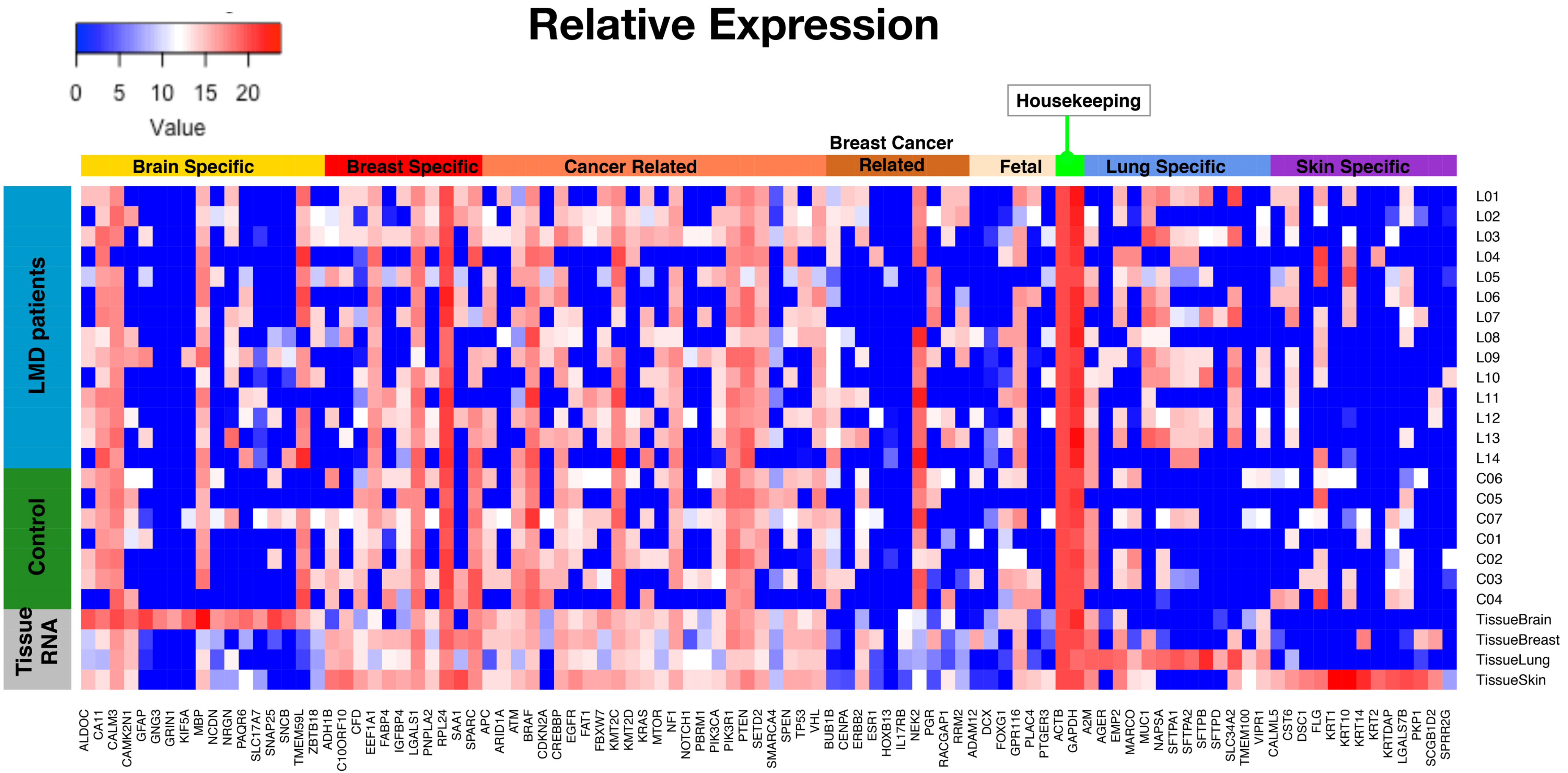

Supplementary Figure 1. This heatmap represents relative expression in all 96 genes tested by high throughput qPCR.

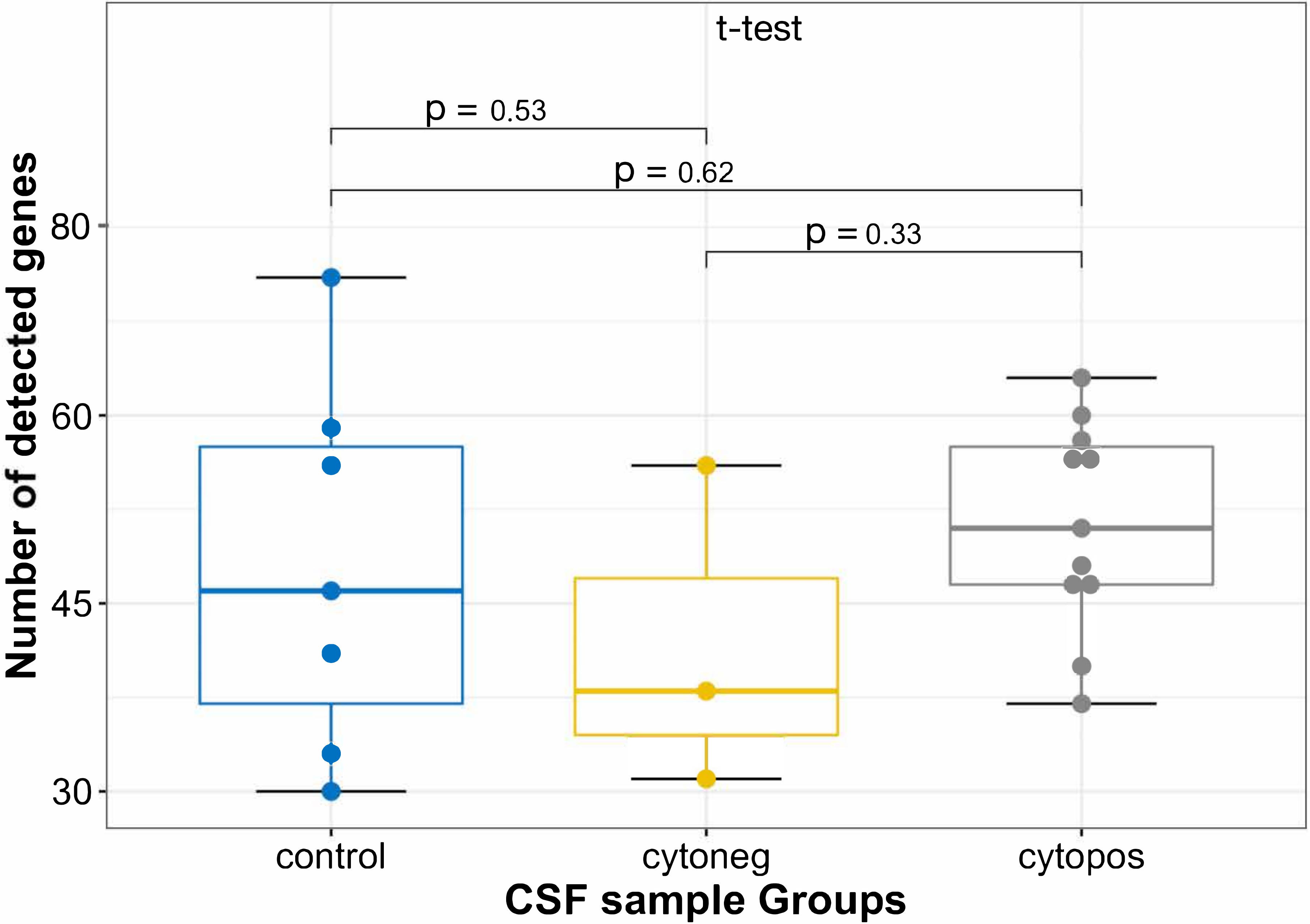

Supplementary Figure 2. This box plot compared the number of detected genes in the parallel qPCR test (total genes =96) among control samples (n=7), cytology negative samples (n=3) and cytology positive samples (n=11). No significant difference was observed by

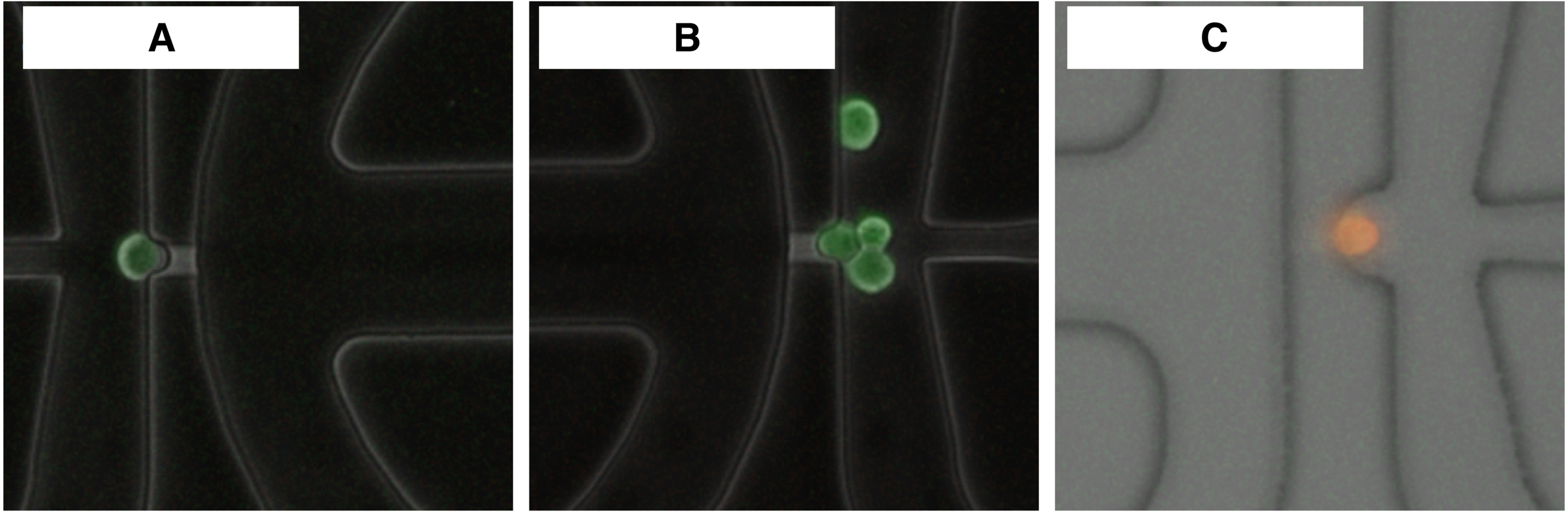

Supplementary Figure 3. Examples of single-cell capture. (A) a live single cell; (B) multiple cells; (C) a dead cell.

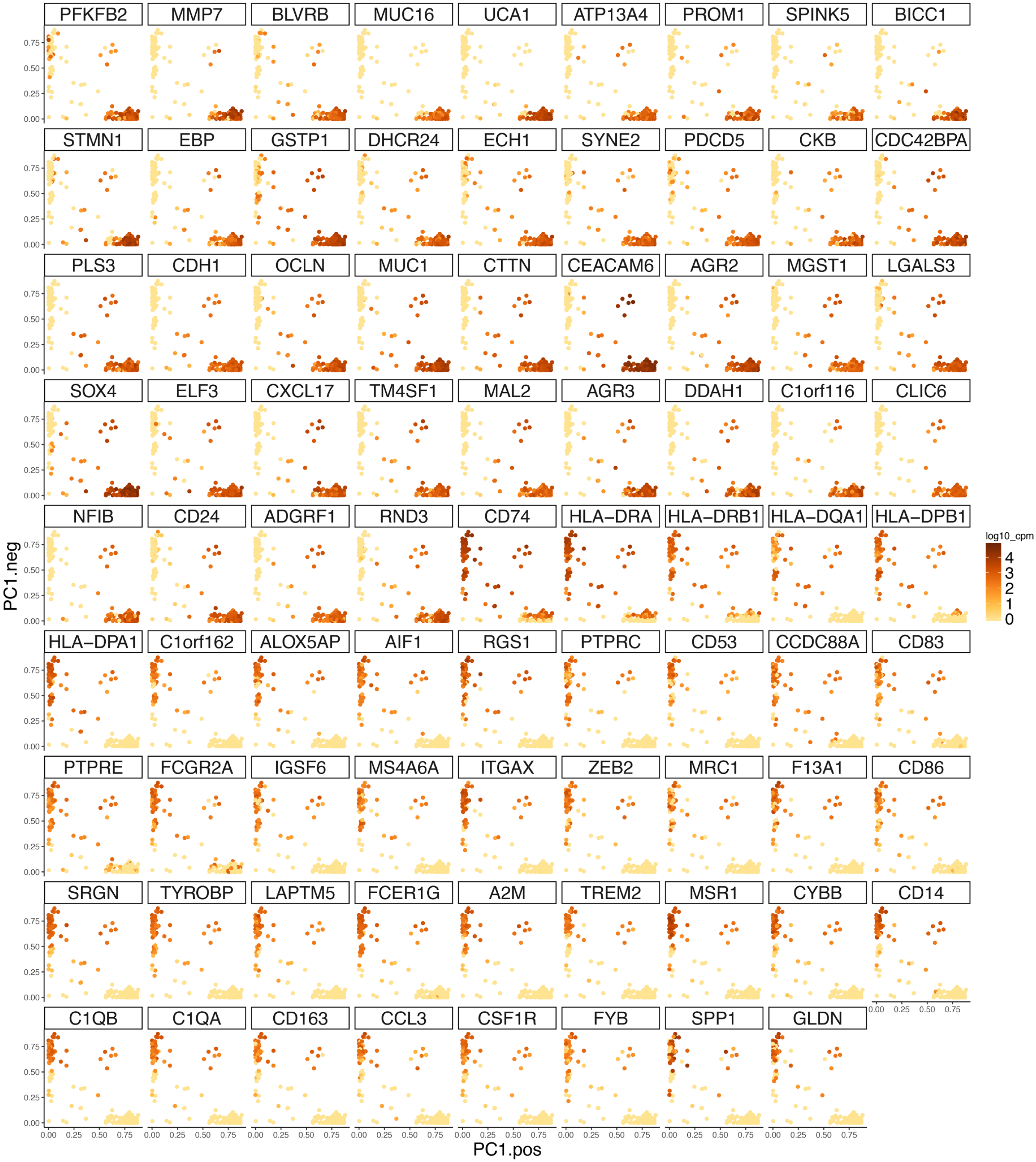

Supplementary Figure 4. Characteristic genes in each cluster from scRNAseq.

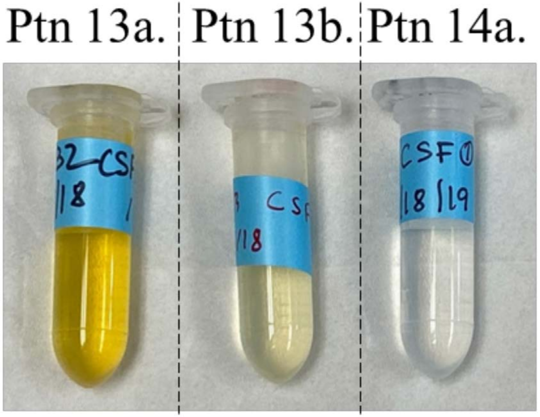

Supplementary Figure 5. Two samples of CSF were collected from patient 13 on different dates. CSF samples Ptn 13a. and Ptn 13b. are tinted with yellow as compared to CSF sample Ptn 14a. The color probably is due to hemolysis residues as this patient suffered from intracranial hemorrhage.

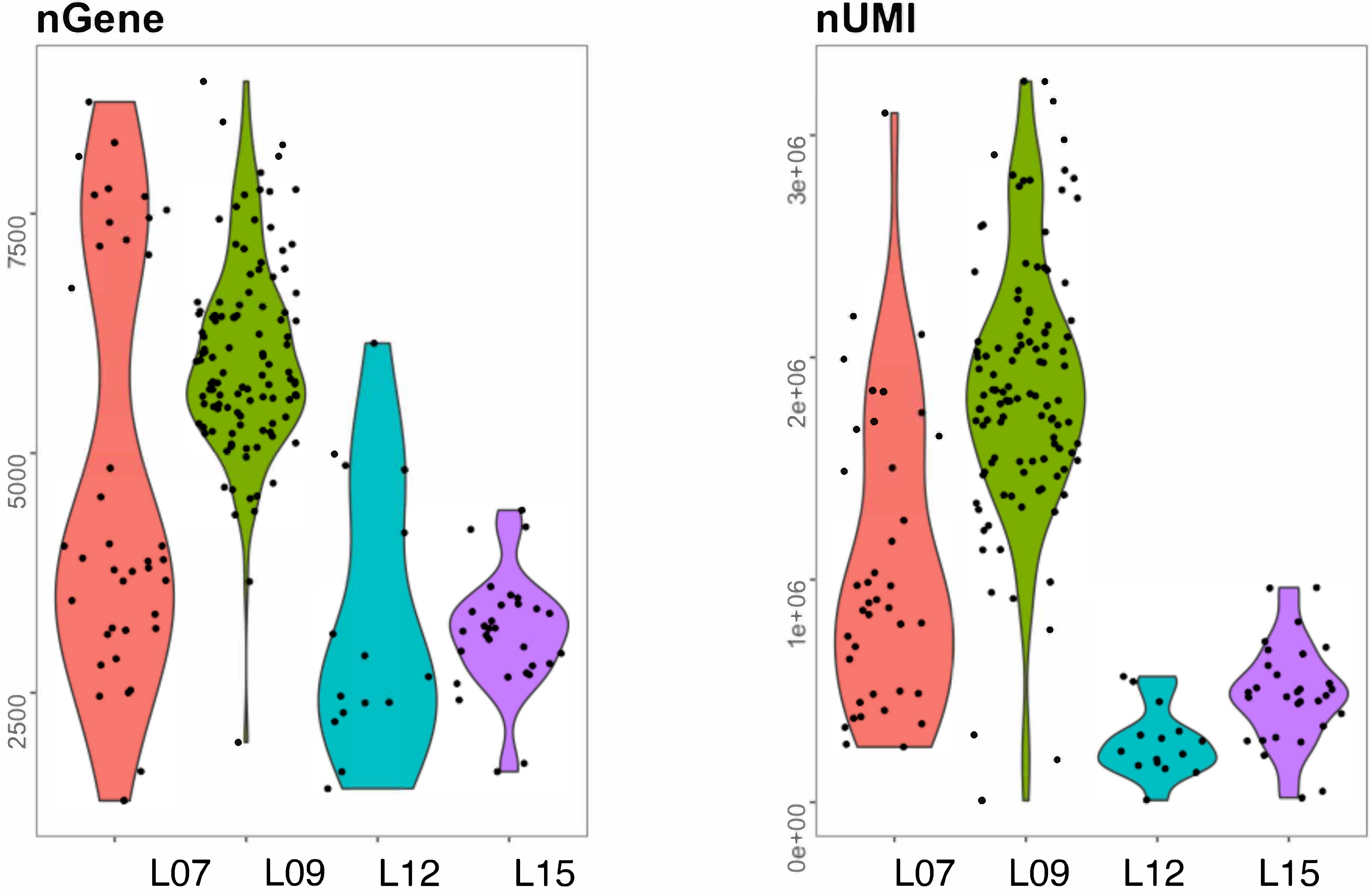

Supplementary Figure 6. Number of genes and number of UMI from scRNA seq.

Supplementary Table 1. Test Cohort - Summary of Clinical Sample Information.

	Sex	Age	Diagnosis	scRNA-seq	Cytology	MRI	Tx before LP	Known mutations
C01	F	44	MVD		NA	NA	NA	NA
C02	M	74	MVD		NA	NA	NA	NA
C03	M	67	MVD		NA	NA	NA	NA
C04	M	62	Pituitary adenoma		NA	NA	NA	NA
C05	F	58	Pituitary adenoma		NA	NA	NA	NA
C06	M	68	Pituitary adenoma		NA	NA	NA	NA
C07	F	43	Pituitary adenoma		NA	NA	NA	NA
L01	F	26	NSCLC		pos	Positive	Radiotherapy, chemotherapy, targeted therapy	Unspecified EGFR mutation
L02	M	65	NSCLC		pos	Positive	Unknown	EGFR exon 19 deletion
L03	M	72	NSCLC		pos	Positive	Targeted therapy	EGFR exon 19 deletion
L04	F	71	NSCLC		neg	Negative	Chemotherapy	EGFR exon 20 mutation
L05	F	43	NSCLC		neg	Suspicious	Radiotherapy chemotherapy, targeted therapy	ROS mutation
L06	M	63	NSCLC		pos	Negative	Stereotactic radiosurgery, chemotherapy, targeted therapy	EGFR 19 deletion
L07	M	58	NSCLC	X	pos	Suspicious	Stereotactic radiosurgery, chemotherapy, targeted therapy, immunotherapy	HER2 mutation

L08	F	72	NSCLC		Suspicious	Suspicious	Stereotactic radiosurgery, chemotherapy, targeted therapy, immunotherapy	None
L09	F	73	NSCLC	X	pos	Positive	Stereotactic radiosurgery, chemotherapy, targeted therapy	TP53 R248W, EGFR 19 deletion
L10	M	84	NSCLC		pos	Positive	Chemotherapy, immunotherapy	EGFR exon 19 deletion
L11	F	47	NSCLC		pos	Suspicious	Stereotactic radiosurgery, targeted therapy	EGFR exon 20 mutation, (pos in urine), EGFR exon 21 mutation
L12	F	40	NSCLC	X	pos	Positive	Chemotherapy, targeted therapy	ALK translocation
L13	F	51	NSCLC		pos	Positive	Stereotactic radiosurgery, chemotherapy, targeted therapy	EGFR exon 21 mutation
L14	F	74	NSCLC		neg	Positive	Chemotherapy, immunotherapy, targeted therapy	EGFR exon 20 mutation
L15	M	72	NSCLC	X	pos	Positive	Targeted therapy	EGFR exon 19 deletion

Supplementary Table 2. Clinical cytology reports of four patients whose CSF underwent single cell RNA sequencing.

Item	Ref. Range	L04	L09	L12		L15
**CSF tube #**	Unknown	4	3	4	1	3
**Color, CSF**	Latest Ref Range: Colorless	Colorless	Colorless	Colorless	Colorless	Colorless
**Turbidity, CSF**	Latest Ref Range: Clear	Clear	Clear	Clear	Clear	Clear
**Color after centrifuge, CSF**	Latest Ref Range: Colorless	Colorless	Colorless	Colorless	Colorless	Colorless
**Turb. after centrif., CSF**	Latest Ref Range: Clear	Clear	Clear	Clear	Clear	Clear
**WBC count, CSF**	Latest Ref Range: 0 - 5 /uL	6 (H)	70 (HH)	2	5	6 (H)
**RBC count, CSF**	Latest Ref Range: 0 - 5 /uL	<1	<1	<1	116 (H)	1
**Neutrophils, CSF**	Latest Ref Range: 0 - 6 %	6	0		2	0
**Lymphocytes, CSF**	Latest Ref Range: 40 - 80 %	3 (L)	24 (L)		71	9 (L)
**Monocytoid cells, CSF**	Latest Ref Range: 15 - 45 %	88 (H)	16		24	91 (H)
**Others. CSF**	Latest Ref Range: 0 %		60 (H)		2 (H)	

Supplementary Table 3. Validation Cohort - Summary of clinical sample information in qPCR validation of CEACAM6

	Sex	Age	Diagnosis	Cytology	MRI	Tx before LP	Known Mutations
**Ctrl 1 "C01"**	F	44	Microvascular Decompression	NA	NA	NA	NA
**Ctrl 2 "C04"**	M	62	Pituitary Adenoma	NA	NA	NA	NA
**Ctrl 3**	F	44	Meningioma	NA	NA	NA	NA
**Ctrl 4**	M	83	Normal Pressure Hydrocephalus	NA	NA	NA	NA
**Ctrl 5**	F	30	Chiari Malformation	NA	NA	NA	NA
**Ptn 1.a**	M	72	NSCLC	Positive	Suspicious	Stereotactic radiosurgery, chemotherapy, targeted therapy	EGFR exon 19 deletion
**Ptn 1.b**	M	72	NSCLC	Positive	Suspicious	Targeted therapy	EGFR exon 19 deletion
**Ptn 2 "L02"**	M	65	NSCLC	Positive	Suspicious	unknown	EGFR exon 19 deletion
**Ptn 3**	F	57	NSCLC	Positive	Positive	Stereotactic radiosurgery, chemotherapy, targeted therapy	EGFR exon 21 mutation
**Ptn 4.a "L07"**	M	58	NSCLC	Positive	Suspicious	Stereotactic radiosurgery, radiotherapy, chemotherapy, targeted therapy, immunotherapy	Not Known
**Ptn 4.b "L07"**	M	58	NSCLC	Positive	Positive	Radiotherapy, chemotherapy, targeted therapy	Not Known
**Ptn 5**	F	75	NSCLC	Positive	Positive	Radiotherapy, chemotherapy, targeted therapy	EGFR exon 20 insertion

	Sex	Age	Diagnosis	Cytology	MRI	Tx before LP	Known Mutations
**Ptn 6**	M	58	Inflammation from PD-1 antibody for NSCLC	Negative	Suspicious	Cranial resection, radiotherapy, chemotherapy, targeted therapy, immunotherapy	Negative
**Ptn 7**	F	62	NSCLC	Positive	Positive	Stereotactic radiosurgery, chemotherapy, immunotherapy	EGFR exon 20 insertion
**Ptn 8 “L13”**	F	51	NSCLC	Positive	Positive	Cranial resection, stereotactic radiosurgery, chemotherapy, targeted therapy	EGFR exon 21 mutation
**Ptn 9**	F	74	NSCLC	Negative	Positive	Chemotherapy, targeted therapy	EGFR exon 20 mutation
**Ptn 10**	F	68	NSCLC	Positive	Positive	Radiotherapy, chemotherapy, targeted therapy, immunotherapy	EGFR exon 19 deletion
**Ptn 11**	M	60	NSCLC	Positive	Positive	Chemotherapy and targeted therapy	EGFR exon 20 and 21 mutations
**Ptn 12 “L15”**	M	72	NSCLC	Positive	Positive	Targeted therapy	EGFR exon 19 deletion
**Ptn 13.a**	F	54	NSCLC	Positive	Positive	Targeted therapy	ALK positive
**Ptn 13.b**	F	54	NSCLC	Positive	Positive	Chemotherapy and targeted therapy	ALK positive
**Ptn 14.a**	M	58	NSCLC	Positive	Negative	Targeted therapy	EGFR exon 21 mutation
**Ptn 14.b**	M	59	NSCLC	Positive	Negative	Chemotherapy, targeted therapy	EGFR exon 21 mutation

	**Sex**	**Age**	**Diagnosis**	**Cytology**	**MRI**	**Tx before LP**	**Known Mutations**
**Ptn 15**	M	59	NSCLC	Negative	Positive	Chemotherapy, targeted therapy, immunotherapy	EGFR exon 19 deletion
**Ptn 16**	M	81	NSCLC	Positive	Positive	Radiotherapy, chemotherapy, targeted therapy, immunotherapy	ALK positive

* a. and b. refer to CSF sample collected from same patient on different dates.
* CSF samples Ctrl 1, Ctrl 2, Ptn 2, Ptn 4, Ptn 8 and Ptn 12 were also used in “test cohort” (Supplementary Table 1).

Supplementary Table 4. Left table - Contingency table describes the correlation between the detection of *CEACAM6* gene in CSF samples and cytology test of LM diagnosis, n=20, $P < 0.01$ (Fisher's exact test). Right table – Sensitivity, Specificity, Positive Prediction Value (PPV), Negative Prediction Value (NPV) and Accuracy parameters to detect *CEACAM6* gene in CSF samples indicative of LM diagnosis.

**Contingency**		
P < 0.01		
	Cytology positive	Cytology negative
CEACAM6 positive	15	0
CEACAM6 negative	2	3

**Sensitivity**	
	%
Sensitivity	88.2
Specificity	100.0
PPV	100.0
NPV	60.0
Accuracy	94.1

Supplementary Table 5. Number of solid parenchymal brain metastases at time of lumbar puncture sample collection.

Pt Number	Cytology	MRI	Parenchymal Brain Mets Y/N	Number of Parenchymal Brain Mets at LP
Ctrl 1 “C01”	NA	NA	NA	NA
Ctrl 2 “C04”	NA	NA	NA	NA
Ctrl 3	NA	NA	NA	NA
Ctrl 4	NA	NA	NA	NA
Ctrl 5	NA	NA	NA	NA
Ptn 1.a	Positive	Suspicious	Y	3
Ptn 1.b	Positive	Suspicious	Y	3
Ptn 2 “L02”	Positive	Suspicious	Y	10
Ptn 3	Positive	Positive	Y	4
Ptn 4.a “L07”	Positive	Suspicious	Y	8
Ptn 4.b “L07”	Positive	Positive	Y	>20
Ptn 5	Positive	Positive	N	0
Ptn 6	Negative	Suspicious	Y	>20
Ptn 7	Positive	Positive	Y	1
Ptn 8 “L13”	Positive	Positive	Y	>20
Ptn 9	Negative	Positive	N	0
Ptn 10	Positive	Positive	Y	4
Ptn 11	Positive	Positive	Y	>20
Ptn 12	Positive	Positive	N	0
Ptn 13.a	Positive	Positive	Y	>20
Ptn 13.b	Positive	Positive	Y	>20
Ptn 14.a	Positive	Negative	N	0
Ptn 14.b	Positive	Negative	N	0
Ptn 15	Negative	Positive	N	0
Ptn 16	Positive	Positive	N	0

* a. and b. refer to CSF sample collected from same patient on different dates.

* CSF samples Ctrl 1, Ctrl 2, Ptn 2, Ptn 4, Ptn 8 and Ptn 12 were also used in “test cohort” (Supplementary Table 1).
