# Supplementary material for: Comprehensive RNA analysis of CSF reveals a role for CEACAM6 in lung cancer leptomeningeal metastases
Source: NPJ Precis Oncol. 2021 Oct 8;5:90. doi: 10.1038/s41698-021-00228-6 (PMC8501028; doi:10.1038/s41698-021-00228-6)
Supplement: Supplementary file 2 — Reporting Summary [file 41698_2021_228_MOESM2_ESM.pdf]

nature research

Corresponding author(s): Dr Melanie Gephart

Reporting Summary

Nature Research wishes to improve the reproducibility of the work that we publish. This form provides structure for consistency and transparency in reporting. For further information on Nature Research policies, see Authors & Referees and the Editorial Policy Checklist.

Statistical parameters

When statistical analyses are reported, confirm that the following items are present in the relevant location (e.g. figure legend, table legend, main text, or Methods section).

n/a	Confirmed	
☐	☒	The exact sample size (*n*) for each experimental group/condition, given as a discrete number and unit of measurement
☐	☒	An indication of whether measurements were taken from distinct samples or whether the same sample was measured repeatedly
☐	☒	The statistical test(s) used AND whether they are one- or two-sided<br>*Only common tests should be described solely by name; describe more complex techniques in the Methods section.*
☐	☒	A description of all covariates tested
☐	☒	A description of any assumptions or corrections, such as tests of normality and adjustment for multiple comparisons
☐	☒	A full description of the statistics including central tendency (e.g. means) or other basic estimates (e.g. regression coefficient) AND variation (e.g. standard deviation) or associated estimates of uncertainty (e.g. confidence intervals)
☐	☒	For null hypothesis testing, the test statistic (e.g. *F*, *t*, *r*) with confidence intervals, effect sizes, degrees of freedom and *P* value noted<br>*Give P values as exact values whenever suitable.*
☒	☐	For Bayesian analysis, information on the choice of priors and Markov chain Monte Carlo settings
☒	☐	For hierarchical and complex designs, identification of the appropriate level for tests and full reporting of outcomes
☒	☐	Estimates of effect sizes (e.g. Cohen's *d*, Pearson's *r*), indicating how they were calculated
☐	☒	Clearly defined error bars<br>*State explicitly what error bars represent (e.g. SD, SE, CI)*

Our web collection on statistics for biologists may be useful.

Software and code

Policy information about availability of computer code

Data collection	Code Availability Statement<br>Analysis script was uploaded to github and can be found using the link below:<br>https://github.com/gmstanle/leptomeningeal-metastases-scRNAseq
Data analysis	FastQC (version 0.11.4; http://www.bioinformatics.babraham.ac.uk/projects/fastqc/) was used for sequencing quality assessment. Reads were then aligned to the human (hg19) transcriptome using Bowtie software (version 2.2.7) with splice junctions being defined in a Gene Transfer Format file (obtained from the University of California, Santa Cruz). Expression at gene level was determined by calculating reads per kilo base per million aligned reads (FPKM) as well as raw count using RSEM software version 1.2.30 (http://deweylab.github.io/RSEM/). Iterative rPCA analysis was conducted using R: A Language and Environment for Statistical Computing version 3.4.1 (R Foundation for Statistical Computing, Vienna, Austria; https://www.R-project.org).

For manuscripts utilizing custom algorithms or software that are central to the research but not yet described in published literature, software must be made available to editors/reviewers upon request. We strongly encourage code deposition in a community repository (e.g. GitHub). See the Nature Research guidelines for submitting code & software for further information.

Data

Policy information about availability of data

All manuscripts must include a data availability statement. This statement should provide the following information, where applicable:
- Accession codes, unique identifiers, or web links for publicly available datasets
- A list of figures that have associated raw data
- A description of any restrictions on data availability

Gene count data can be found at the link https://figshare.com/account/home#/projects/78399.
In addition, data will be added to the publicly available website: www.LMDseq.org.
The non-sequencing data and materials are available from the corresponding author on reasonable request.
Gene count data can be found at the link https://figshare.com/account/home#/projects/78399.
In addition, data will be added to the publicly available website: www.LMDseq.org.
The non-sequencing data and materials are available from the corresponding author on reasonable request.

Field-specific reporting

Please select the best fit for your research. If you are not sure, read the appropriate sections before making your selection.

☒ Life sciences ☐ Behavioural & social sciences

For a reference copy of the document with all sections, see nature.com/authors/policies/ReportingSummary-flat.pdf

Life sciences

Study design

All studies must disclose on these points even when the disclosure is negative.

Sample size	Human CSF samples were collected from patients with or without cancer (controls) through a Stanford University Institutional Review Board-approved protocol. As a proof of concept case series, sample availability depended upon patient consent, volume obtained, and quality of the sample; patients were not specifically recruited for this study. LM CSF samples were obtained either from a standard-of-care lumbar puncture (LP) or ventriculostomy. Patients either required CSF access for diagnosis (e.g. equivocal MRI and/or concerning symptoms of LM), or therapeutic treatment of increased intracranial pressure. Only CSF samples in excess of what was required for clinical pathological diagnosis were utilized in this study; as a pilot trial, no additional, invasive procedures were performed.
Data exclusions	no data were excluded from the analysis.
Replication	Western blot, qPCR and cellular assays were done with technical (3) and biologic replicates (2-3).
Randomization	For patient-derived samples the groups were assigned by diagnosis, tumor vs. non-tumor samples.
Blinding	At the time of samples collection, in most cases, the scientist didn't know the diagnosis and often not at the moment of sample processing.

Materials & experimental systems

Policy information about availability of materials

n/a	Involved in the study	
☐	☒	Unique materials
☒	☐	Antibodies
☒	☐	Eukaryotic cell lines
☒	☐	Research animals
☐	☒	Human research participants

Unique materials

Obtaining unique materials	Patients' CSF samples are unique in this study and unfortunately for each such sample were consumed to conduct the study.

Human research participants

Policy information about studies involving human research participants

Population characteristics	We provide extensive de-identified clinical and demographic information pertinent to the collected and tested patients' samples in this study.

Method-specific reporting

n/a	Involved in the study
☒	☐ ChIP-seq
☒	☐ Flow cytometry
☒	☐ Magnetic resonance imaging
